# A comprehensive eHealth implementation guide constructed on a qualitative case study on barriers and facilitators of the digital care platform CMyLife

**DOI:** 10.1186/s12913-022-08020-3

**Published:** 2022-06-06

**Authors:** Lynn Verweij, Yolba Smit, Nicole MA Blijlevens, Rosella PMG Hermens

**Affiliations:** 1 Department of Hematology, Radboud Institute for Health Sciences, Radboud University Medical Center, Nijmegen, Netherlands; 2 Department of IQ Healthcare, Radboud Institute for Health Sciences, Radboud University Medical Center, Nijmegen, Netherlands

**Keywords:** Implementation guide, Barriers, Facilitators, Digital care platforms, CMyLife, Patient empowerment, Qualitative case study

## Abstract

**Background:**

Substantial proliferation of eHealth has enabled a move in patient-centred cancer care from the traditional in-person care model to real-time, dynamic, and technology supported on-demand care. However, in general, the uptake of these innovations is low. Studies show that eHealth is helpful in providing patient empowerment through e.g. providing high quality and timely information, enabling self-monitoring and shared decision making, but dropout rates are high and guidance for optimal implementation is lacking.

**Aim:**

To explore barriers to and facilitators for nationwide implementation and consolidation of CMyLife, a multi-component, patient-centred, digital care platform, and to construct a comprehensive implementation guide for launching digital care platforms in daily clinical practice.

**Methods:**

The first qualitative case study of a digital care platform like CMyLife was performed including five focus group- and eighteen in-depth interviews with stakeholders. Data were collected using a semi-structured interview guide, based on the frameworks of Grol and Flottorp. Transcripts of the interviews were analysed and barriers and facilitators were identified and categorized according to the frameworks. An iterative process including participation of main stakeholders and using the CFIR-ERIC framework led to creating a comprehensive implementation guide for digital care platforms.

**Results:**

In total, 45 barriers and 41 facilitators were identified. Main barriers were lack of connectivity between information technology systems, changing role for both health care providers and patients, insufficient time and resources, doubts about privacy and security of data, and insufficient digital skills of users. Main facilitators mentioned were motivating patients and health care providers by clarifying the added value of use of a digital care platform, clear business case with vision, demonstrating (cost) effectiveness, using an implementation guide, and educating patients and health care providers about how to use CMyLife. Based on these barriers and facilitators a clear and comprehensive implementation guide was developed for digital care platforms.

**Conclusion:**

Several barriers to and facilitators for implementation were identified, a clear overview was presented, and a unique comprehensive implementation guide was developed for launching future digital care platforms in daily clinical practice. The next step is to validate the implementation guide in other (oncological) diseases.

**Supplementary Information:**

The online version contains supplementary material available at 10.1186/s12913-022-08020-3.

## Background

Substantial proliferation of eHealth has enabled a move in patient-centred cancer care from traditional in-person care model to real-time, dynamic, and technology supported on-demand care [1]. An example of a basic form of eHealth is an accessible electronic medical record which provides patients with personal health information in a secured digital environment through a webbased portal [2, 3]. In this era of advanced digital technologies, eHealth is also able to offer more than just a single component [4]. One of the most extensive eHealth innovations is a digital care platform (DCP) [5]. A DCP provides patients with multiple valuable components, such as personalized education about their condition and their treatment, an overview of their personal health record, including medical results and appointments, leading to better information provision. It also offers patients a place for direct messaging with other patients and secure patient-provider messaging, leading to better communication between patients and their health care providers (HCPs) [6–8]. Additionally, it may allow patients to register patient-reported outcome measurements to monitor and manage adverse events, leading to better medication compliance [9]. Use of aforementioned, more advanced, multicomponent DCPs has shown to empower patients and help to incorporate them as an equal member of their own care team [4, 6].

Despite all these benefits, and shown helpfulness in improving care of many patients, uptake of these innovations is low and studies reported high dropout rates [10–13]. Guidance for optimal implementation of eHealth is lacking and the first step to creating this is investigating barriers to and facilitators for implementation of eHealth. Previous studies have shown that there are many reasons for patients and their HCPs to use or not use eHealth. For instance, patient needs may change over the course of their disease, and different patients prefer different types of support [14]. Innovations are more likely to be used and to be successful when they provide targeted and timely support including information relevant to a specific group [15]. Involving end users during the development of eHealth showed to increase the likelihood of the technology actually being used [2, 10, 16]. Besides, implementation of eHealth often involves complex organisational change and thereby has a major impact on health care organisation [17].

A specific group of patients in which eHealth has shown to be very important are oncological patients, for example by providing better medication management, patient empowerment, and information provision [5, 18, 19]. A recent review showed that several studies evaluated barriers to and facilitators for implementation in oncological care [5]. However, these studies mostly included limited, rather simple eHealth innovations, and had limited diversity of involved stakeholders. Previous studies by Kooij et al. (2018), Cremers et al. (2021), and Stanimirović and Vintar (2014) did evaluate barriers to and facilitators for DCP implementation, but they did not differentiate between simple forms of eHealth and more advanced multi-component innovations, they did not include stakeholders from within and outside the hospital organisation, or they did not base their results on a specific practical example [20–22]. Nevertheless, it is not clear what is needed for nationwide implementation of a multi-component DCP and little is known about appropriate specifications of such a platform in oncological care [5, 23]. Perspectives of other stakeholders with an organisational, economic and political background from outside the hospital organisation are also important to be evaluated, for example, health insurers who can provide insight in what is required to realize structural and sustainable financing of eHealth [5]. Since there is no one size fits all, the next generation of eHealth systems should develop and refine health applications acknowledging the complex and changing needs of not only patients but all stakeholders involved, from both within and outside the hospital organisation.

CMyLife is an example of a more advanced, multi-component DCP that provides patient-centred care through empowering patients which could lead to the opportunity of hospital-free care for patients with chronic myeloid leukemia (CML). The development, features, and effectiveness of the CMyLife platform are described in detail elsewhere [24]. CML is a chronic, life-long malignant disease characterized by translocation of chromosome 9 and 22, leading to the BCR-ABL1 mutation, which encodes for a constitutively active tyrosine kinase [25, 26]. Treatment consists of daily oral medication (tyrosine kinase inhibitors) and if CML patients have optimal responses to the treatment their life expectancy approaches that of the general population [27, 28]. Therefore, the primary aim of CMyLife is to improve medication compliance and monitoring of the biomarker BCR-ABL1 because these factors determine treatment success [29].

Throughout the development of CMyLife the lack of implementation guidance in the field of DCPs in general, and in oncology specifically, was felt. For example, challenges in differing end user- and stakeholder needs, and behavioural and organisational resistance were encountered throughout the development and implementation phases of the various components of the DCP. The literature outlined that these issues were not specific to our context, but more universal in nature, as described above. Therefore, the research question of this study was: “What are barriers to and facilitators for nationwide implementation and consolidation of CMyLife, a multi-component, patient-centred, DCP, and which implementation activities, combined in a comprehensive implementation guide, cover these barriers?”

## Methods

### Design

A qualitative study including focus group- and in-depth interviews with stakeholders involved in the implementation of the CMyLife platform was performed to explore barriers to and facilitators for nationwide implementation and consolidation of CMyLife. Study participants for the interviews were recruited between August 2018 and October 2020 and included different stakeholders to reach successful implementation [30]. After the interviews, a comprehensive implementation guide for DCPs was developed in an iterative process with involvement of the multidisciplinary CMyLife project group and a national expert panel. This study was reported in accordance with the STARi and COREQ checklists [31, 32].

### Setting

In the Netherlands CML is treated in eight academic hospitals and in 68 smaller peripheral hospitals. The Dutch-Belgian Cooperative Trial Group for Hematology-Oncology is established to monitor the quality of haematological care. The latest CML guidelines recommend that molecular treatment response should be measured at least every three months in the first year, and every four to six months thereafter [33]. Since CML is a chronic disease patients' biomarker levels are monitored during their entire life. CML medication falls under the Dutch expensive medicine regulation and can only be prescribed by haematologists. The Dutch health care system involves mandatory health care insurance, covering all CML care which makes treatment accessible to all patients. Since CML is a rare disease treatment results differ between hospitals with high and low CML patient numbers receiving treatment [34].

The multidisciplinary CMyLife project group was formed to coordinate the process and consisted of representatives of the main stakeholders: patients, haematologists, specialized nurses, molecular biologists, and pharmacists. The DCP CMyLife is described in Table 1.

**Table 1:** Description of DCP CMyLife

**Fig. 1:**
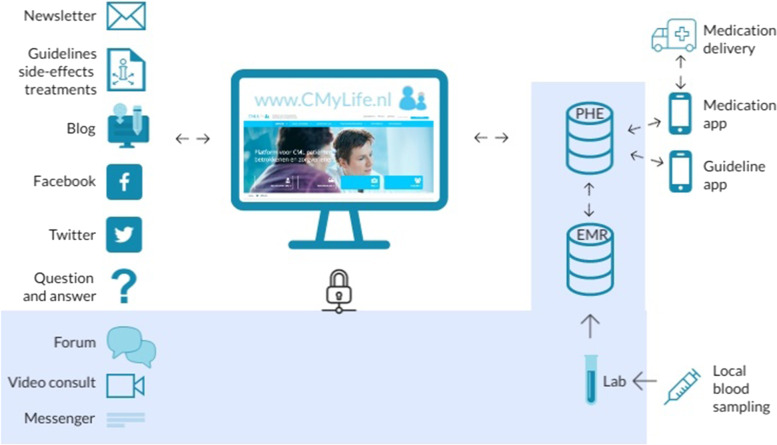
The CMyLife platform and its components. PHE= personal health environment, EMR=electronic medical record, all components depicted in the blue box were secured with a two-step validation with a token received via an SMS text message

The development and features of the CMyLife platform were described in detail elsewhere [24]. CMyLife facilitates CML patients with a website (www.cmylife.nl), a medication app, a guideline app, and a personal health environment (PHE). The CMyLife platform and its components are depicted below (Fig. 1).
The **website** provides accurate and easy to understand information about CML, medication, guidelines, side effects, and the effect on daily life (work, sports, mortgage etc.). Through the website patients are enabled to communicate with specialists and other patients.
The **medication app** [35] is used to set medication alarms, register their medication intake, request for repeat medication prescriptions and read the information leaflet of medication. In addition, patients can log the side effects they experience, which can be shared with their HCPs through their PHE.
The **guideline app** [33] enables self-monitoring of the biomarker BCR-ABL1, by sending monitoring reminders according to the Dutch CML guideline, and shows an understandable explanation of patients’ BCR-ABL1 values in relation to the Dutch guideline.
Patients can save their own medical records from their electronic medical record in their **personal health environment,** consisting of a Patient Knows Best portal [36]. For example, they can share their side effects with their HCP in order to discuss them.

### Study participants

Purposeful sampling was applied and stakeholders were approached face-to-face, via telephone, or via email to participate in this study [37]. In total, 23 interviews were performed of which five were focus group interviews with four to sixteen stakeholders of the main stakeholder groups and eighteen were individual interviews with each at least two of the additional stakeholders. Table 2 shows an overview of the amount of participants in the (focus group) interviews. Main stakeholder groups consisted of the multidisciplinary project group, patients, haematologists, specialized nurses, and molecular biologists. The project group consisted of a policy researcher, a specialized nurse, a molecular biologist, the polyclinical pharmacist, a patient advocate of Hematon (the Dutch patient advocate association), the CMyLife project leader and the CMyLife community manager. Regarding the focus groups with haematologists and molecular biologists, researchers joined existing meetings, therefore more participants were present. The other focus groups were specially organised.

**Table 2:** Overview of study participants

**Focus group interviews**	**N**	**Individual interviews**	**N**
Project group	7	Employees of pharmaceutical companies	2
Patients	6	Employees of the Netherlands comprehensive cancer organisation	2
Haematologists	16	IT-specialists	3
Specialized nurses	4	Health insurers	2
Molecular biologists	10	Policy makers	2
		Privacy officers	2
		Pharmacists	5

### Data collection

**Fig. 2:**
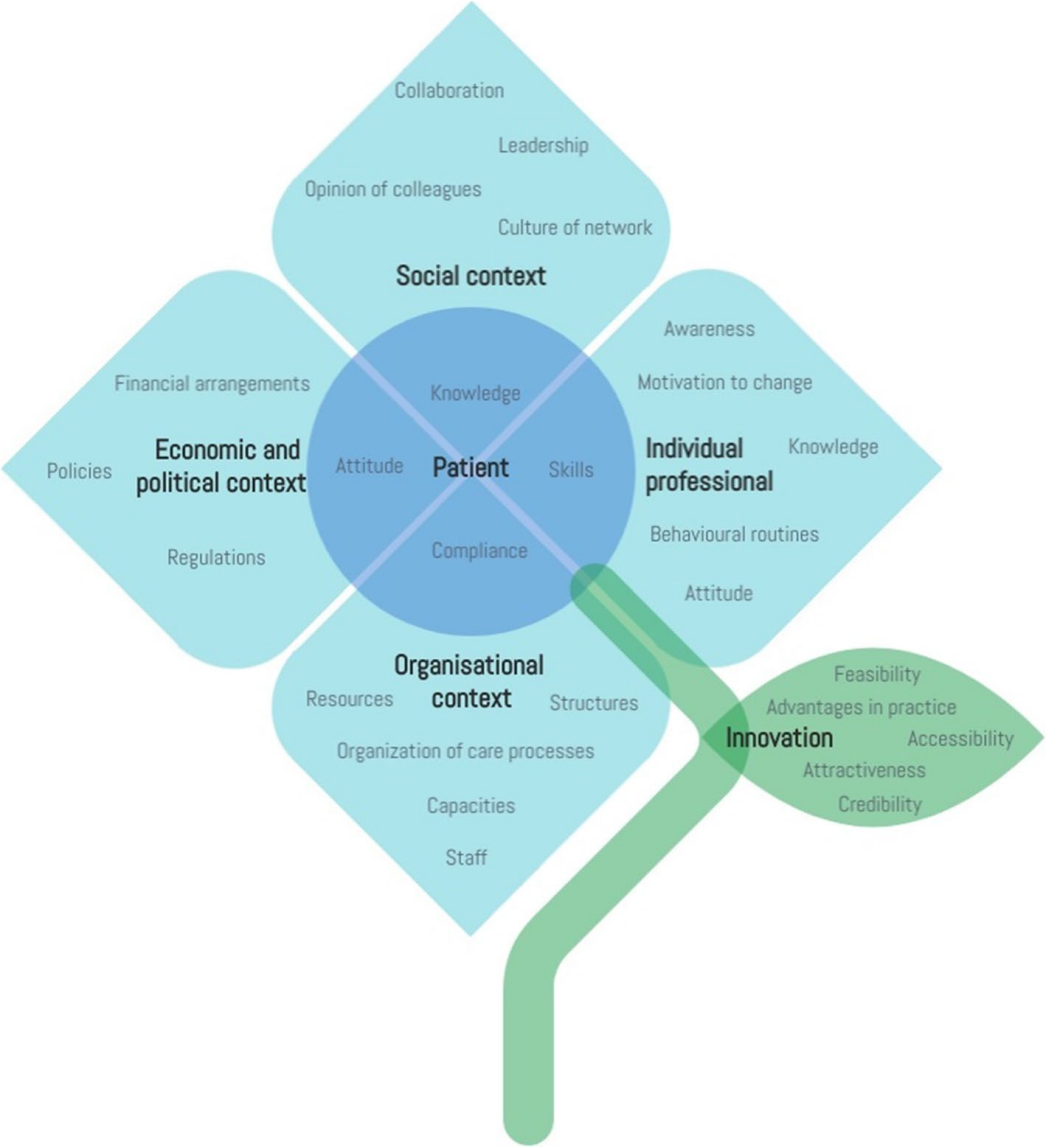
Barriers to and facilitators for implementation at levels of healthcare [38, 39]

Data on barriers and facilitators were collected using a semi-structured interview guide, based on the frameworks of Grol and Flottorp [38, 39], defining the barriers to and facilitators for change in healthcare practice at 6 different levels: (1) the innovation itself; (2) patient; (3) individual professional (4) organisational context; (5) social context; and (6) economic and political context. Figure 2 shows a visual of the applied framework. The semi-structured interview guide was used to ensure that the main questions were asked in every interview and to have sufficient freedom to expand and ask for additional clarification on specific answers and thereby gaining in-depth understanding of how barriers and facilitators could influence implementation. Additional file 1 shows the interview guide translated from Dutch. The (focus group) interviews were held on location, which the participants preferred, or by telephone if requested, by three different interviewers (IM, RH and SR) (researchers with much experience in qualitative research) independently. No power relation between the interviewers and the interviewees was expected, therefore this did not affect the answers of the interviewees during the interviews. Informed consent was obtained before the start of each interview.

At the start of the interviews, interviewees were asked for their consent to make audio recordings of the interviews. Then, participants were informed about the background of the interviewer and CMyLife, the current situation of the platform, the duration of the interview, and the purpose of the interview. After this, the interviewees were asked about their expected or perceived barriers to and facilitators for the nationwide implementation and consolidation of CMyLife using the above mentioned semi-structured interview guide. After the interview, the interviewer gave a general summary of the interview and checked whether someone had any additional information. Finally, participants were thanked for their participation. In addition to the interviews, literature and reports about barriers to and facilitators for implementation were checked for additional barriers and facilitators for implementation.

### Data analysis

First, all interviews were transcribed and all data from the interviews were anonymized. Transcripts of the interviews were qualitatively analysed by two researchers (IM and LV). They independently coded text fragments that reflected a barrier to or a facilitator for implementation of CMyLife, and then categorized them according to the above mentioned frameworks using the software programme Atlas.ti [38]. Since a pre-existing model was used for coding, the approach was deductive [40, 41]. After independently coding the transcripts, the codes were discussed between the two researchers until consensus was reached. In case of a remaining discrepancy, a third researcher was asked to arbitrate (RH). An overview of all identified barriers to and facilitators for the nationwide implementation and consolidation of CMyLife was created. To illustrate the meaning of the barriers and facilitators, quotations that were considered representative are reported in the results section. Quotations were derived from the (focus group) interviews and translated from Dutch. Creation of the implementation guide is described in detail below.

### Creation of implementation guide

**Fig. 3:**
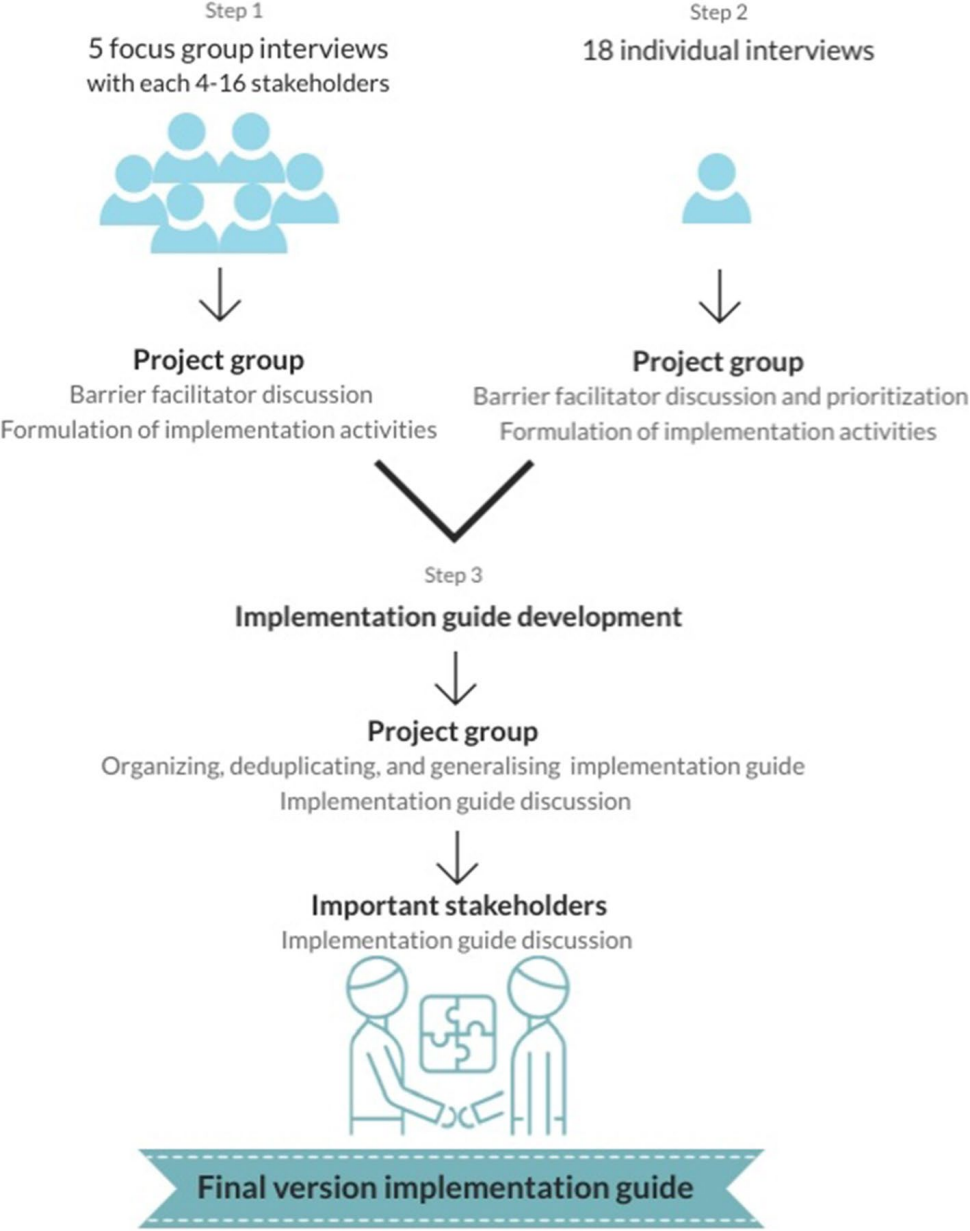
Summary of steps from interviews to implementation guide

An iterative process including participation of the main stakeholders in the multidisciplinary CMyLife project group led to creating a comprehensive implementation guide for launching DCPs in daily clinical practice (Fig. 3). This process was guided by the CFIR-ERIC (Consolidated Framework for Implementation Research-Expert Recommendations for Implementing Change) Implementation Strategy Matching tool, evaluating current barriers and facilitators for implementation and matching potential actions (strategies) using facilitators to reduce barriers [42]. First, barriers and facilitators gathered from the focus group interviews were shared and discussed. For each barrier, project group members proposed one or several potential solutions, mostly based on facilitators, and formulated matching implementation activities. Then, after the individual interviews the barriers and facilitators were again shared and discussed with the project group. They were asked if they recognized these factors and if there were any missing barriers or facilitators. Next, all gathered barriers and facilitators were plenary discussed, prioritized, and linked to matching implementation activities by the project group. Prioritization was based on what was needed to have (priority 1) versus nice to have (priority 2), and in part on what is absolutely necessary to start with (priority 1) and what can be added during the process of implementation (priority 2). Priority 3 were barriers that should be taken care of but this is beyond the control of the team implementing a DCP. Implementation experts (RH and LV) developed an implementation guide including implementation strategies using the CFIR-ERIC tool, on advise of The Netherlands Organisation for Health Research and Development [42]. After organising, deduplicating, and generalising the first draft of the implementation guide, it was once again shared and discussed with the multidisciplinary project group and subsequently with the most important stakeholders (patients, haematologists, specialized nurses and molecular biologists and pharmacists) in a digital expert panel meeting to come to a final version.

## Results

### Interview characteristics

In total, 61 stakeholders were interviewed in order to identify barriers to and facilitators for the nationwide implementation and consolidation of CMyLife. Twenty-three different interviews were performed of which eighteen were individual in-depth interviews and five were focus group interviews. The (focus group) interviews lasted between 12 and 81 minutes.

### Barriers to and facilitators for implementation of CMyLife

In total, 910 quotes were selected and 45 barriers to and 41 facilitators for implementation of CMyLife were identified. Table 3 shows an overview of all identified barriers to and facilitators for the nationwide implementation and consolidation of CMyLife, prioritization of the barriers and if barriers and facilitators were integrated in the implementation guide (in blue). Results are presented according to the six levels of the frameworks used. Barriers and facilitators in need of more explanation or barriers with first priority are presented in the text below accompanied by quotes to illustrate and clarify the barriers and facilitators. For example, the meaning of the barrier “too disease specific” is not entirely clear and needs some clarifying explanation.

**Table 3:**
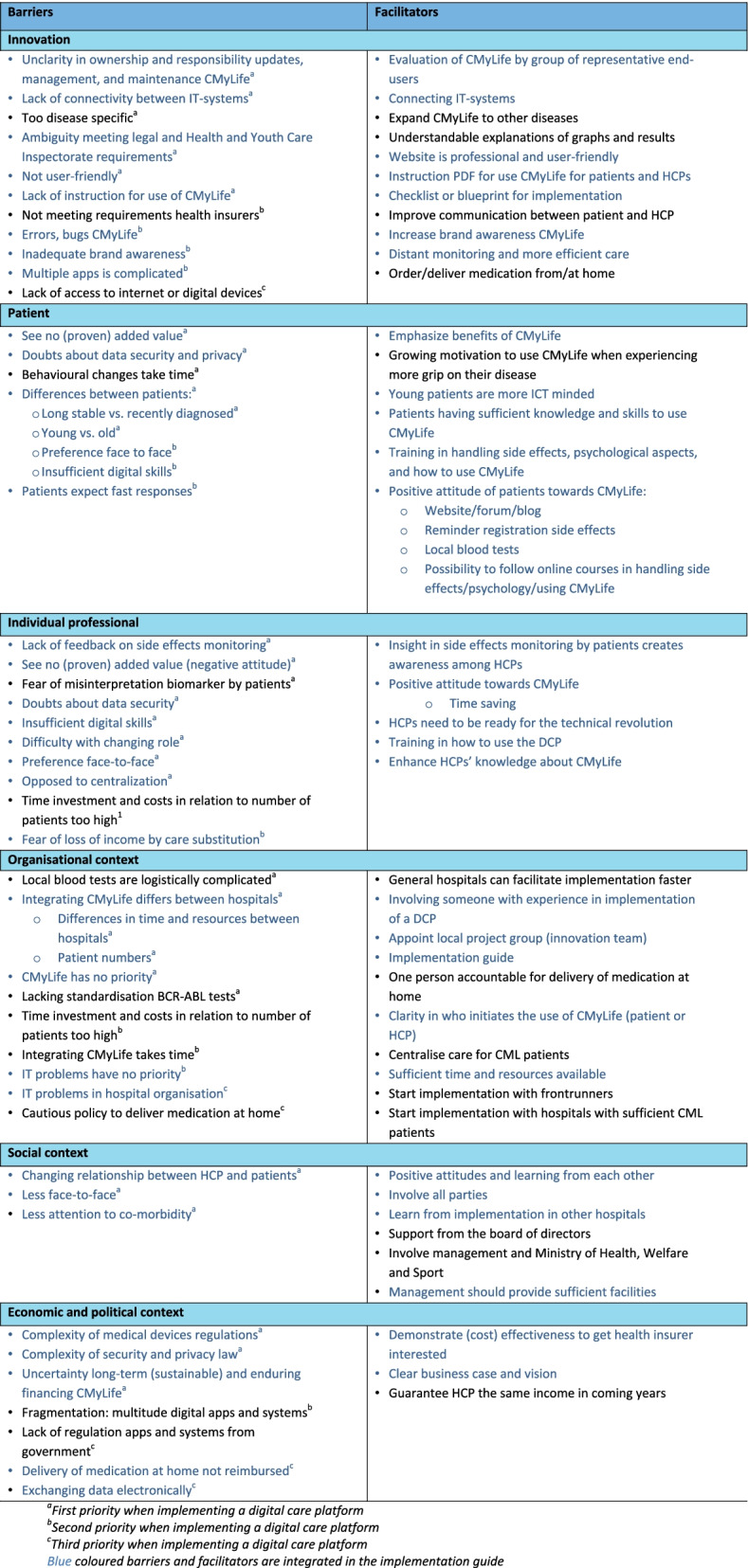
Overview of barriers to and facilitators for the nationwide implementation and consolidation of CMyLife

### Innovation: CMyLife

#### Barriers

Lack of connectivity between IT-systems, the innovation being too disease specific, unclarity in ownership and responsibility updates, management, and maintenance of CMyLife, and aspects of CMyLife not being user-friendly were identified as barriers on innovation level. CMyLife consisted of several systems which were not all interconnected, this hindered dataflow between systems and users of the platform had to fill in information multiple times. The innovation was only focussed on patients with CML. This is a small patient population and some hospitals only treat a few of these patients. Also, there was no clarity about who was responsible for updates, management and maintenance of CMyLife. Stakeholders also mentioned that some aspects of the innovation were not user-friendly. For example, patients mentioned that the frequency of registering side effects was too high and when they suffer from specific side effects over a longer period, they do not want to register the same side effects repeatedly. The login to CMyLife was ‘a lot of hassle’, patients should login multiple times (because of the lack of connectivity between IT-systems) in different ways and it did not always work.

“You should think about who is eventually owner of CMyLife and who takes care of updates and who keeps the platform going.“ (IT-specialist)

“Receiving a reminder for registering side effects only once a week would be better. Like, take a look back at your week which side effects did you suffer from, I would actually do that.” (Patient)

#### Facilitators

CMyLife enabling patients to receive more efficient care and improving the communication between patients and their HCPs were mentioned as facilitators for implementation. HCPs were positive about the use of CMyLife, they agreed that the use of CMyLife made care more efficient, it saved them time and decreased waiting and travel time for patients. Patients knew their biomarker values beforehand and were therefore able to prepare better for their consultation. Therefore, CMyLife improves the communication between HCPs and patients. Another facilitator mentioned on innovation level was the availability of a checklist, blueprint or implementation guide before implementation, including information about connecting IT-systems, privacy and security, step-by-step what should be arranged and in what order.

“The biomarkers were good and the app shows that the patient took his medication well, yes, who are we to say that they should also physically see that patient, that is not necessary at all.” (Health insurer)

### Patient

#### Barriers

Doubts about data security and privacy, patients not seeing a (proven) added value and differences between patients were barriers mentioned on patient level. Several stakeholders mentioned that patients feared that their personal data may end up publicly exposed. Also, differences between patients were often mentioned as a barrier to implementation. For example, using CMyLife was not a necessity for patients who were stable over a long period of time, however using CMyLife could have a great added value for patients who were recently diagnosed with CML. In addition, young patients had generally better digital skills and were more open to using DCPs compared to older patients.

“I think that contact with my healthcare professional is important, so I just visit four times a year.” (Patient)

#### Facilitators

The possibility to follow online courses in handling side effects, psychological aspects, and how to use CMyLife and patients having a positive attitude towards CMyLife were mentioned as facilitators for implementation on patient level. Patients mentioned to be pleased with the attractiveness and the usefulness of the website, the forum and the blog. Also, they liked to receive a reminder to register their side effects and they preferred to have their blood tested locally. Testing blood locally saved them waiting and travel time.

“I think it is possible to make sure that patients have their blood drawn at home, or somewhere near their house, and patients could only come for a consultation when their medication or biomarkers factor are not ok. This way both travel time and office hours can be saved. I think win-win-win.” (Health insurer)

### Individual professional

#### Barriers

Among others, barriers mentioned on individual professional level were insufficient digital skills and difficulty with the changing role of HCPs. Stakeholders also mentioned that time investment and costs in relation to number of patients was too high. The role of both the HCP and the patient was changing, adjusting to this new role will take time and effort. However, work pressure and understaffing hampered this process. Another mentioned barrier on individual professional level is that some HCPs preferred face-to-face communication. They wanted to prevent missing crucial things about patients’ lives and possible comorbidities. Also, older HCPs tended to value personal contact more and had less digital skills.

“How interesting is your work if there is no more personal contact. It is not only important to hear about side effects but also the story around it.” (Haematologist)

“Since work pressure is high and changes like this take time it is hard to get everyone cooperative with implementation.” (Haematologist)

#### Facilitators

HCPs who were enthusiastic and positive about CMyLife and who clearly understood the usefulness of CMyLife facilitated the implementation of CMyLife. It was important to enhance HCPs’ knowledge about the benefits of the use of CMyLife and how to work with DCPs. Training HCPs in the use of DCPs was also mentioned as a facilitator for implementation.

“Make the advantages that CMyLife has very prominent for everyone, then it seems almost impossible not to support it as a centre.” (IT-specialist)

“Keep informing people what you are doing and keep them engaged, motivation and information is essential.” (Specialized nurse)

### Organisational context

#### Barriers

Barriers on organisational context level were numerous. Time investment and costs in relation to number of patients was mentioned to be too high. Integrating CMyLife differed between hospitals, with some hospitals having enough time and resources and others not having this advantage. General hospitals were less involved with innovation and development compared to academic hospitals. Also, IT problems in the hospital organisation were a barrier to implementation, such as bad internet connection. Most of the time IT problems did not have priority in hospitals, it took time to fix these problems and good IT employees were scarce.

“I think the biggest problem is IT.” (Specialized nurse)

#### Facilitators

Involving someone with experience during implementation of DCPs, appointing a local project group, using an implementation guide, and centralizing care were facilitators mentioned on organisational level. The implementation guide should be some sort of blue print or instructional manual about what aspects the innovation should contain and a step-by-step plan for implementation. Someone with experience in implementing the innovation could help to implement it in other hospitals and a project group including all involved stakeholders facilitated implementation.

“You have to create a project group with the important parties in your hospital and they should lead implementation.” (Project group)

### Social context

#### Barriers

On social context level the changing relationship between HCPs and patients, including less face-to-face contact and less attention for co-morbidity were mentioned as barriers to the implementation of CMyLife. HCPs should allow patients to take more control over their disease and care will be more from a distance. Also, not all HCPs agree with centralization of care for CML patients because this meant that some of them lose their CML patients.

“What I'm concerned about is that if you're going to centralize the care, some people have to let go of something and they don’t want to.” (Employee IKNL)

#### Facilitators

Positive attitudes and learning from others were mentioned as facilitators for implementation on social level as well as involving all stakeholders during the process to create support. Support from colleagues, informing each other, sharing experiences and learning from each other will make a big difference in the acceptance of DCPs. Also, learning from implementation in other hospitals was mentioned as a facilitator for implementation.

“I think that raising awareness and taking our colleagues along will be the first challenge to make it land properly with other hospitals.” (Project group)

“Seeing good examples up close, that can motivate, to exchange and discuss this with each other, to really go a little deeper, to stimulate each other.” (Policymaker)

### Economic and political context

#### Barriers

The complexity of medical devices regulations, the complexity of security and privacy law, the uncertainty of long-term (sustainable) and enduring financing and the lack of regulation of apps and systems by the government were mentioned as barriers to the implementation of CMyLife. Because personal data is handled electronically in these kind of innovations the complexity of the security and privacy law was an important barrier. Before implementation all laws should be taken into account carefully. Also, connectivity between IT-systems was a challenge because of these laws.

“It would be a shame if continuity is at stake because gaps in financing arise, sponsors might disappear at some time, then it will become very unstable, you should not want that, it would be nice if it became wider supported, also by health insurers that have an interest in it, but perhaps also clinics that can step in, but then there must also be financial benefits in return.” (Pharmaceutical company)

“Before we can implement at all, I think everything around privacy and security must be arranged properly.” (IT-specialist)

#### Facilitators

A clear business case, vision, and demonstrating (cost) effectiveness to get health insurers interested were mentioned as facilitators for implementation on economic and political context level. CML patients are a vulnerable patient group, health insurers indicated that this is an important fact to mention in a conversation about sustainable financing and getting support from health insurers.

“The moment we see initiatives that improve patient care and it has a positive effect on costs, we are interested to play a role.’’ (Health insurer)

“For health insurers it is very important that there is a business case behind your innovation, that is crucial for these kinds of conversations.” (Health insurer)

### Implementation guide

To take care of the identified barriers to implementation and use the identified facilitators for implementation an implementation guide was composed. Based on main themes returning in the interviews the implementation guide was divided in different phases of DCP implementation; development of the innovation, dissemination, continuous motivation and support, the context (here the hospital-specific context), and actual implementation and long-term and enduring financing DCP. Eventually, a clear but comprehensive implementation guide was developed for DCPs. Table 4 shows the activities of the implementation guide in combination with the type of strategy according to the CFIR-ERIC framework.

**Table 4:** Activities of the implementation guide for digital care platforms in combination with the strategy

**Phase of implementation**	**Strategy**
**Development innovation**	
Formulate clear goal, vision, focus, and strategy	Innovation optimizing strategy
Establish a clear organisational and governance structure	
Create a clear overview of IT-system architecture	
Make data infrastructure and data flow transparent	
Ensure that the innovation complies with regulations regarding data security and privacy	
Set a clear business case	
Set a change plan and/or continuously adjust innovation based on change plan	
Give platform/apps appropriate name(s)	
Involve key stakeholders from the start during development of the innovation	Product focussed strategy
Work intensively with end users on content, design, further development/improvement of the innovation	Innovation optimizing and product focussed strategy
Work on (further) development via short cyclical improvement, e.g. the so-called plan do check act cycle	
In the (further) development of the innovation, take diversity of patients into account	
Get the technical aspects in order and set up structure for solving bugs	
Minimize the number of different IT parties involved	Innovation optimizing strategy
Make concrete agreements with IT party(ies) about responsibility for updates and security	Cooperation promoting strategy
Involve IT/privacy officer continuously in the design, further development and improvement of the innovation	
Explore (cost-)effectiveness of the DCP	
**Dissemination**	
Communicate vision, focus, strategy, organisational & governance structure, system architecture, data infrastructure & data flow	Informative strategy
Communicate agreements with IT party(ies) about responsibility for updates and security with relevant stakeholders	
Promote innovation through patient conferences	
Find and use additional distribution channels that are already working well	
Communicate (cost-)effectiveness	
**Continuous motivation and support of end users**	
Evaluate user experiences of the innovation and provide insight into effectiveness, ensuring that stakeholders/users will see the added value of the platform	Motivation and support increasing strategy
Engage key figures and opinion leaders to provide motivation & support for innovation among stakeholders/users to increase	
Have project team members (or rather the key figures) go to hospitals for personal approach & highlighting the benefits of DCPs	
Organise meetings with patients & healthcare providers (focus groups, workshops, conferences) for support	
To motivate caregivers for change (to see the need)	
Make the content of the innovation attractive to end users. For example, use an up-to-date website so that the usefulness of the DCP becomes clear	
**Hospital-specific context**	
In terms of IT, make sure that the 'unwieldy hospital equipment' is circumvented as much as possible so the DCP does not/barely needs to link with hospital systems	Organisational strategy
Ideally, there is a connection between electronic patient files and the innovation (when relevant)	
Inform and motivate hospital organisations about the importance of the innovation for patients; provide a fixed point of contact in the hospital who feels responsible for a smooth implementation	Informative and motivation and support increasing strategy
Emphasize that good IT facilities for implementation of the innovation are a must have for hospitals	
**Actual implementation and long-term and enduring financing DCP**	
Prepare and set a good blueprint/guideline for the rollout of the innovation, including the involvement of stakeholders; among other things, deal with the IT connectivity of systems, privacy & security, step-by-step plan with what needs to be arranged in a hospital before implementation and in what order, and where to go for support	Facilitating strategy
Start implementation on a small scale	
As a project team, offer temporary support for implementation; for the long term a good business model is indispensable	
Provide training for caregivers, if necessary (do not focus on the ultimate user-friendly tool because this is different for everybody) work with smart PDF including instructions	Educational strategy
When implementing with patients, provide extra support for certain groups	Motivation and support increasing strategy
Ensure the use of patient-reported outcome measurements data during a consultation, when patient-reported outcome measurements management is part of the DCP	
Integrate the DCP into the clinical pathway as much as possible	
Ensure assurance in 3 steps: start with the idea, continue to exploit innovation, build a good business case (with real-world evidence!) and obtain financing for long-term and enduring financing	Market-oriented strategy

## Discussion

This study presented the first qualitative case study of a DCP like CMyLife on barriers to and facilitators for nationwide implementation among various stakeholders within and outside the hospital organisation, which led to a comprehensive implementation guide for DCPs. In total, 45 barriers to and 41 facilitators for implementation of CMyLife were identified. Main barriers mentioned were a lack of connectivity between IT-systems, a changing role for both HCPs and patients, insufficient time and recourses, doubt about privacy and security of data, and insufficient digital skills of patients and their HCPs. Main facilitators mentioned were motivating patients and their HCPs by clarifying the added value of the use of the DCP, a clear business case, vision, and demonstrating (cost) effectiveness, using an implementation guide, and educating patients and HCPs about benefits and use of CMyLife. By using the barriers and facilitators we developed a comprehensive implementation guide for launching DCPs in daily clinical practice.

Kooij et al. (2018) identified barriers to and facilitators for DCP implementation facing various stakeholders within the hospital organization, thereby had a narrower scope compared to our study [20]. Also, Kooij et al. (2018) [20] reported differences in the included hospitals with regard to the features of the concerned DCP and the phase of the DCP. Some hospitals had already provided a DCP, while others were in the middle of the implementation process or had no DCP at all. This could have caused different results from our study because expectations before implementations and the experiences afterwards can vary among HCPs and patients [43, 44]. In our study the DCP already existed and stakeholders were familiar with its components [24]. Despite these differences between our study and the study by Kooij et al. (2018) [20], results are quite comparable. For instance, concerning barriers to implementation, lack of time and resources and guaranteeing privacy and security were mentioned as well. Concerning the facilitators for implementation, positive attitudes, and management support (strategy plan for implementation) were also mentioned. However, our study found additional barriers and facilitators, for example, the study by Kooij et al. (2018) [20] did not find barriers on individual professional level and facilitators on patient level whereas our study did. This is probably because they included stakeholders from hospitals with and without an implemented patient portal, this could have introduced bias into responses because some participants identified barriers and facilitators without even using a DCP and therefore had to imagine the implementation process. Like our study, Kooij et al. (2018) [20] indicated that having an implementation guide would be a facilitator for implementation. The current study provides this practical guide for the implementation of DCPs. How translatable are the results of our CMyLife DCP, specifically developed with and for CML patients, to DCPs in general? Barriers and facilitators which are only applicable for CMyLife, for example that the platform is too disease specific (because of the small patient population) and lacking standardization of BCR-ABL1 tests are not used in the implementation guide. CMyLife comprises all features of a DCP as described in the introduction. Therefore, our developed implementation guide can be widely used for other DCPs including these features, not only national but results also seem applicable internationally. Nevertheless, the Dutch health organisation may differ from other countries, for example in universal health care coverage. This may involve differences in barriers and facilitators. To our knowledge this is the best (retrospective) display of the barriers and facilitators for nationwide implementation of CMyLife.

In a previous study by Cremers et al. (2021) an eHealth implementation guideline was developed as well, using a literature search and a two-round Delphi study among experts [21]. They presumed eHealth in general and did not differentiate between simple forms of eHealth and more advanced multi-component innovations. Contextual factors matter, there is no one size fits all and implementation guides for these digital tools will differ since implementation of a DCP is more complicated compared to implementation of single component eHealth. Our study developed an implementation guide for DCPs in particular and besides literature, stakeholder and expert opinions were based on a specific practical example. Differences between results may be caused by these differences between the studies. For example, the study by Cremers et al. (2021)  [21] states that for a successful implementation suitable patients or patient groups should be identified to participate in the eHealth program based on predefined inclusion and exclusion criteria. Our study aims to provide all interested patients with suitable education and training to make them able to use the innovation and continue using it, as can be seen from the implementation phase; continuous motivation and support in the implementation guide. In addition to the differences between the results of the studies, results are also comparable between studies. They also mention that eHealth implementation is very complex and a variety of variables should be taken into account.

This complexity regarding national success factors in the development of eHealth interventions was also stressed by the study by Stanimirović and Vintar (2014) [22]. Their study identified the main deficiencies in eHealth implementation, they mapped a set of general success factors in the field and suggested guidelines for the effective development and implementation of eHealth projects. Again this study did not differentiate between simple forms of eHealth and a more advanced multi-component innovation, such as a DCP. For this reason results between their study and our study are not entirely comparable. For example they state that political commitment to reform and reorganisation of the clinical departments are success factors for effective development and implementation of eHealth. These factors are not mentioned in our implementation guide because they are beyond the control of the team implementing a DCP. Still, there are some agreements with our study. For example, inclusion of stakeholders and effective collaboration and promoting the use of IT, education and training.

Our study comprised several strengths and limitations. The use of semi-structured interview guides provided the interviewers with the opportunity to learn the reasons behind the answers. Semi-structured interviews allow interviewees to open up about sensitive issues and since three different interviewers performed the interviews, the use of semi-structured interviews ensured that main questions were consistently asked in every interview and participants were given freedom to exceed on asked questions. Therefore, the use of semi-structured interviews did not restrict stakeholders in thinking outside the box when answering the questions. In our study, six patients were interviewed and 30 healthcare workers. This might have raised concerns that this influenced the results of the study in a way that the opinions of the healthcare workers were more prominently presented. However, this was not the case since this qualitative study focussed on quality and not quantity of results. The opinions of all different types of stakeholders were equally analysed (as described in the methods section). In addition, our study is the first study exploring barriers and facilitators in stakeholders from within and outside the hospital organisation, which adds insight into both perspectives.

In order to make sure that this exploration of barriers to and facilitators for the nationwide implementation of CMyLife will lead to quality improvement of the platform, it is important that the content of CMyLife gets improved accordingly and expanded to other cancer types. Future research is required to determine whether the implementation guide is actually useful in the implementation of a DCP and to determine the validity of this implementation guide in other (oncological) diseases. In addition, it needs to be validated and recalibrated regularly to increase its potential to facilitate implementation and the next step after successful implementation is investigating what is a necessity to establish sustained use of DCPs among patients and their HCPs.

## Conclusion

The aim of the present research was to explore barriers to and facilitators for the nationwide implementation and consolidation of the CMyLife platform, leading to a comprehensive implementation guide for launching DCPs in daily clinical practice. This research has shown that the nationwide implementation of a DCP is a complex process. Main barriers were lack of connectivity between information technology systems, changing role for both HCPs and patients, insufficient time and resources, doubts about privacy and security of data, and insufficient digital skills. Main facilitators mentioned were motivating patients and HCPs by clarifying the added value of use of the DCP, a clear business case with vision, demonstrating (cost) effectiveness, using an implementation guide, and educating patients and HCPs about the use of CMyLife. Based on these barriers and facilitators a strategic and comprehensive implementation guide was developed as this study and previous studies asked for. Future research should be undertaken to confirm the practical utility and explore whether this implementation guide actually facilitates implementation of DCPs.

## Supplementary Information

**Additional file 1.** Interview guide CMyLife. 

## Data Availability

The datasets used and/or analysed during the current study are available from the corresponding author on reasonable request.

## Abbreviations

CML: Chronic Myeloid Leukemia
DCP: Digital Care Platform
HCP: Health Care Provider
CFIR: Consolidated Framework for Implementation Research
ERIC: Expert Recommendations for Implementing Change
