# Supplementary material for: A comprehensive eHealth implementation guide constructed on a qualitative case study on barriers and facilitators of the digital care platform CMyLife
Source: BMC Health Serv Res. 2022 Jun 6;22:751. doi: 10.1186/s12913-022-08020-3 (PMC9169388; doi:10.1186/s12913-022-08020-3)
Supplement: Supplementary file 1 — Additional file 1. Interview guide CMyLife. [file 12913_2022_8020_MOESM1_ESM.pdf]

Interview guide CMyLife e-Clinics

Barriers and facilitators for the national implementation and consolidation of CMyLife

1. Format focus group interviews CMyLife

Background CMyLife platform
CMyLife has two main goals:

- Give patients control of their CML care; this requires optimal patient information
- Organize hospital free care

Why put ‘control in the hands of the patients’?

- The patient can then ensure that his/her blood levels are checked on time (and receives his/her blood result on time).
- Patient knows how to act after receiving his/her blood results.
- Patient receives feedback on his medication intake (insight into the relationship between his/her medication intake and his/her blood value.
- Patient can make his/her complaints known and receive feedback on them.
- Patient is provided with all the information that the care provider wants to provide him/her.

How is that organized**:** with the help of digital tools (website CMyLife, medication app & guideline app, bcr-abl charts and personal health environment), feedback from healthcare providers and the CMyLife support desk for patients and healthcare providers.

Why hospital-free care?

- Patient is free to visit the hospital.

How is that organized: process change in each hospital via local blood tests, delivery of medication, video consultation by the hospital’s specialized nurse.

Situation at the Radboudumc
In the Radboudumc CMyLife e-clinics has already been partially implemented (website & apps are available, local blood tests, delivery of medication and video consultation (now also by haematologists) are possible).
However, implementation of CMyLife e-clinics in other Dutch hospitals will not happen automatically. A first step to realize implementation in other Dutch hospitals is to make an inventory of barriers and facilitators among people who have contributed to the implementation at Radboudumc, the CMyLife project group. This inventory takes place with the use of a focus group interview and is carried out in the context of the ZonMw action research.

Structure and purpose of the focus group interviews
As indicated above, we initially organize a focus group discussion with the CMyLife project group. This will allow us to get a better picture of the experiences with the implementation of CMyLife e-clinics and of possible barriers and facilitators for implementation. The goal is to hear as many experiences as possible without striving for consensus or saturation. In addition to the group discussion with the project group, the project members who cannot attend the focus group discussion will be interviewed separately (a second focus group may be planned). The previous inventory of 'stakeholders' who have a role in national implementation, from the project group meeting on the 11th of June, is supplemented from the group discussion and the additional discussions. In a second interview round, focus group and/or individual interviews are planned with them.

2. Performance interviews

Practical issues

- Arrange space or room to perform the interviews
- Create+sent invitation letter for participants
- Create+sent infromation to participants (important to have a factsheet about the apps)
- Working (recording) equipment
- Arrange and fill out Informed consent forms
- Provide short questionnaires on participants background characteristics, pens and paper to create nametags.

Points of attention in advance

- The interviewer must have a good idea of the distribution of the topics in her/his head;
- During the conversation it is important to verify whether it is the opinion of a respondent and possibly reflect on the aforementioned opinions;
- When a participant does not express his opinion or to a lesser extent, the interviewer tries to stimulate a respondent with previously inventoried opinions.
- Asking questions, trying to understand the participant's perspective;
- Do not defend or explain;
- Listen to whether the respondent answers the question, be alert;
- Do not move on to the next topic too quickly, but take breaks after a participant has said something;
- Ask for additional information. Example questions can be:
  o Can you explain that further?
  o Can you give me an example of what you mean?
  o What else can you say about it?
  o Tell us more?
  o Is there anything else?
  o Would you like to describe what you mean?
  o I don't quite understand
  o Are there any other insights?

FOCUS GROUP TOPICS

- Broad: barriers and facilitators of the national implementation of CMyLife e-clinics based on the 6 domains of the Grol & Wensing model (innovation itself, individual professionals, patient, social setting, organizational context, economic & political context (financing or legislation & regulations)
- Zoom in on: the significance of CMyLife e-clinics for the way of working as a healthcare provider
- Zoom in on: the significance of CMyLife e-clinics on the relationship and communication with the patient
- Zooming in on: the attitude of healthcare professionals and how to influence
- Zoom in on: dealing with digital skills of patients and healthcare professionals

3. Protocol focus group CMyLife e-clinics

WELCOME + THANK FOR PARTICIPATION + ASK PERMISSION FOR AUDIO RECORDING

INTRODUCTION

- Introduction of our research concerning CMyLife e-clinics
- Emphasize confidentiality and voluntariness, as well as confidentiality of recording and data processing
- Propose to tutor each other during the interview and whether there is any objection to this
- - Time indication, mention break
- Are there any questions before we start?

INDIVIDUAL INTRODUCTION

Introductory question

CMyLife is largely implemented at the Radboudumc. The next step is implementation in other hospitals, on the way to national implementation and consolidation. If you look at that, what barriers do you expect? What are factors that can facilitate implementation?

We will discuss this on the basis of the 6 domains of the Grol & Wensing framework (innovation itself, individual professional, patient, social setting, organizational context, economic & political context (financing or legislation & regulations). your first reaction to this question?

With regard to CMyLife, by this we mean all parts, so both the parts needed for ‘patient in control’ and the parts needed for 'hospital-free care’.

TOPICS

 <u>Topic: CMyLife / eHealth apps</u>

Which barriers do you expect, in relation to the implementation of CMyLife in other hospitals, ***in the field of CMyLife, the innovation itself***?

- What barriers have you experienced at Radboudumc in the field of innovation itself?
- What barriers do you expect in other centers? Do you expect differences between the different types of hospitals (UMCs, large peripheral, small peripheral)?

What are facilitators for the implementation of CMyLife in other hospitals, ***in the field of CMyLife, the innovation itself?***

- What is working well in the Radboudumc?
- Does this apply to CMyLife as a whole? Or does this apply to certain parts?

Examples:

- Accessibility of CMyLife
- Attractiveness of CMyLife
- Ease of use of CMyLife (clear, easy to master, operation)
- To what extent CMyLife is consistent with daily routines
- Credibility of CMyLife content
- To what extent CMyLife can be useful for work
- To what extent CMyLife can contribute to productivity
- What is needed (what needs to change) in the area of innovation itself to facilitate implementation in other hospitals?

 Topic: Individual professional

Which barriers do you expect, in relation to the implementation of CMyLife in other hospitals, ***in the field of individual professional?***

- What barriers have you experienced at Radboudumc in the field of innovation itself?
- What barriers do you expect in other centers? Do you expect differences between the different types of hospitals (UMCs, large peripheral, small peripheral)?

What are facilitators for the implementation of CMyLife in other hospitals, ***in the field of individual professional***?

- What is working well in the Radboudumc?
- Does this apply to CMyLife as a whole? Or does this apply to certain parts?

Examples:

- Knowledge regarding the implementation of an e-health innovation
- Attitude towards the implementation of an e-health innovation, e.g. do you expect to use it?
- Motivation regarding the implementation of an e-health innovation, e.g. What is your prediction that you will use CMyLife in the coming months?

What is needed (what needs to change) in the field of the **individual professional** to facilitate implementation in other hospitals?

 Topic: Patient

Which barriers do you expect, in relation to the implementation of CMyLife in other hospitals, ***in the field of the patient?***

- What barriers have patients experienced in the Radboudumc?
- What barriers do you expect in other hospitals for patients?

What are facilitators for the implementation of CMyLife in other hospitals, ***in the field of the patient***?

- What is working well in the Radboudumc?
- Does this apply to CMyLife as a whole? Or does this apply to certain parts?

Examples:

- Patient knowledge about using an app
- Patients' skills to use an app
- Patients' attitudes towards an app
- To what extent is CMyLife understandable for patients

What is needed (what needs to change) in **the patient field** to facilitate implementation in other hospitals?

 Topic: Social setting

Which barriers do you expect, in relation to the implementation of CMyLife in other hospitals, ***the social setting area***

- What barriers did you experience in the social setting area in the Radboudumc?
- What barriers do you expect in other hospitals in the field of social setting?

What are facilitators for the implementation of CMyLife in other hospitals, ***in the social setting area***?

- What is working well in the Radboudumc?
- Does this apply to CMyLife as a whole? Or does this apply to certain parts?

Examples:

- Opinion of colleagues; do you expect people whose opinions you value to think you should start using CMyLife?

- Culture within the organization; Do you expect your employer/colleague(s) to be/are helpful when using CMyLife?
- Cooperation

What is needed (what needs to change) in **the social setting area** to facilitate implementation in other hospitals?

 Topic: Organisational context

Which barriers do you expect, in relation to the implementation of CMyLife in other hospitals, ***in organisational context***?

- What barriers did you experience in organisational context in the Radboudumc?
- What barriers do you expect in other hospitals in organisational context?

What are facilitators for the implementation of CMyLife in other hospitals, ***in the field of organisational context***?

- What is working well in the Radboudumc?
- Does this apply to CMyLife as a whole? Or does this apply to certain parts?

Examples:

- Organization of care processes
- Staff
- Resources
- Need for support
- Differences between different healthcare institutions

What is needed (what needs to change) in **the organization context** to facilitate implementation in other hospitals?

 Topic: economic and political context

Which barriers do you expect, in relation to the implementation of CMyLife in other hospitals, ***in economic and political context***?

- What barriers did you experience in economic and political context at the Radboudumc?
- What barriers do you expect in other hospitals in economic and political context?

What are facilitators for the implementation of CMyLife in other hospitals, ***in economic and political context***?

- What is working well in the Radboudumc?
- Does this apply to CMyLife as a whole? Or does this apply to certain parts?

Examples:

- Financial arrangements
- Laws and regulations
- Policy
- ownership

What is needed (what needs to change) in **economic and political context** to facilitate implementation in other hospitals?

 Topic: Significance of CMyLife for the way of working as a caregiver

Question: what does CMyLife mean for the work and way of working?

For example, think of:

- How a doctor or nurse takes notes
- Additional tasks

- Discuss it with colleagues
- Difference with colleagues
- Consequences: quality of care, efficiency, financial, communication, patient management, control and transparency of information?
- Change of conversation between you and the patient, added value?

- Topic: Significance of CMyLife on patient relationship and communication
  Question: what does CMyLife mean for patient communication?

- Topic: Significance of CMyLife for the attitude of healthcare professionals and how to influence
  Question: what attitude is needed by healthcare professionals for the use of CMyLife and how can this be influenced?
- Topic: Significance of using CMyLife for the digital skills of patients and healthcare professionals
  Question: What does the use of CMyLife require from the digital skills of patients and healthcare professionals?

4. Closing the (focus group) interview

- General summary + check if someone has a reaction or addition
- Last question: did we miss something?
- Thankyou fort he input!
- Complete the interview

→Turn of recording equipment!
